# Agentic artificial intelligence in medicine and biomedical research: A primer and framework for clinicians and researchers

**DOI:** 10.1016/j.jdin.2026.06.001

**Published:** 2026-06-11

**Authors:** Jonathan Kantor

**Affiliations:** a Department of Engineering Science, University of Oxford, Oxford, UK; b Department of Biostatistics, Epidemiology, and Informatics, Perelman School of Medicine at the University of Pennsylvania, Philadelphia, PA; c Florida Center for Dermatology, PA, St Augustine, FL

**Keywords:** Agentic artificial intelligence, artificial intelligence agents, artificial intelligence governance, artificial intelligence hallucination, artificial intelligence oversight, artificial intelligence safety, authorship, automation bias, compounding errors, deskilling, error propagation, human-in-the-loop, ironies of automation, large language models, medical education, medical errors, patient safety, peer review, scientific publishing

## From revolution to regime change

The rise of chatbot-based generative artificial intelligence (AI) systems has been hailed as a revolution in medicine, and these systems have shown widespread use cases ranging from differential diagnosis support to manuscript editing. Yet the pivot to agentic AI—systems that can iteratively pursue goals through multistep processes rather than simply generating an isolated output in response to a single prompt—suggests that this next phase of AI implementation represents an escalation from revolution to true regime change.

**Fig 1:**
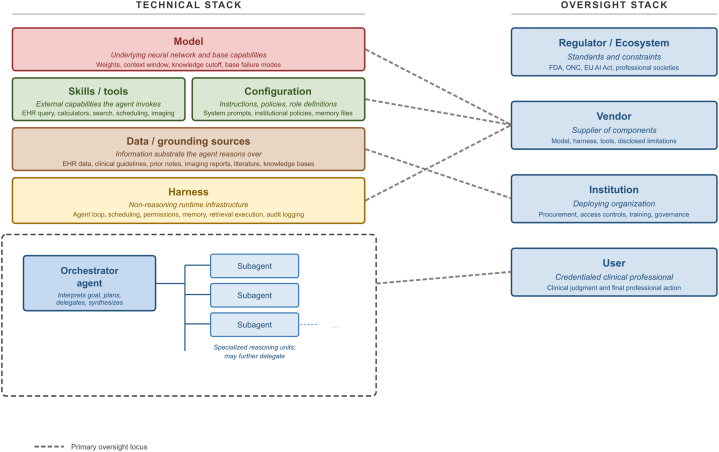
Technical and oversight stacks for agentic AI in clinical and biomedical research settings. *AI*, Artificial intelligence; *EU*, European Union; *FDA*, food and drug administration; *ONC*, Office of the National Coordinator for Health Information Technology.

Agentic systems can plan tasks, use tools, retrieve information, monitor intermediate results, revise their approaches, loop, and execute complex iterative workflows with minimal (or no) human oversight (Table I). Unlike chatbot interactions, which represent a single-inference event, agentic system outputs may be the result of dozens, hundreds, or thousands of such events whose individual content the user often never sees. The supervisory relationship is thus no longer one of inspecting an answer and engaging in hallucination scanning; it is one of trying to govern a workflow, even after the final complex output may have already been created by the system. Agentic AI entails a categorical shift in both how AI interacts with the world and how it can(not) be supervised. AI laboratories are readying for this new reality,^1^ but it is one for which the clinical, regulatory, and methodological literature is largely unprepared (Fig 1).

**Table I:** Glossary of key terms in agentic AI

Term	Definition
Agent	An operational system in which a model is embedded within a harness and given defined tools, permissions, configuration, and task objectives. In multiagent deployments, the clinically relevant agent may be the entire topology, including orchestrator and subagents, rather than a single reasoning entity.
Agentic AI	The broader paradigm of systems that pursue goals through multistep, adaptive action rather than producing a single-inference output in response to a single prompt. This includes planning, tool use, (semi)autonomous execution, and looping.
Agent loop	The iterative control structure through which an agent perceives the current state, reasons about next steps, acts through tools or subagents, observes the result, and revises its plan.
Agent topology	The structural arrangement of reasoning entities within a deployed agent system. A topology may involve a single agent, an orchestrator with subagents, hierarchical delegation, or peer-to-peer multiagent interaction.
Configuration artifact	Pre-written natural-language file injected into context to shape agent behavior. Includes system prompts, persona definitions, project-specific instructions (CLAUDE.md, AGENTS.md), and identity or memory files (SOUL.md, MEMORY.md).
Context window	A model-layer property; the maximum number of tokens the model can process in a single-inference call. A constraint on what the model can attend to at once, distinct from longer-term persistence handled by the harness.
Engineered memory	Harness-layer sub-component providing persistence across invocations: vector stores, structured databases, summarization layers. Distinct from the model's context window, which resets at each invocation.
Grounding source	The external information substrate used to constrain or inform agent reasoning, such as EHR data, clinical guidelines, full-text literature, institutional policies, imaging reports, registries, or knowledge bases. Grounding quality affects the validity of downstream agent outputs.
Harness	The runtime infrastructure that operates the agent. The harness manages the agent loop, context assembly, tool calls, scheduling, permissions, retrieval, memory, logging, and handoffs among agents. The harness does not itself reason, but it strongly shapes what the model can do.
Heartbeat	Harness-layer sub-component; the temporal control structure that determines whether an agent operates reactively (invoked when prompted) or proactively (running on schedule).
Model	The underlying neural network with its weights, base capabilities, and failure modes. The model generates reasoning outputs, but it does not act outside the harness and tool environment. Context window and knowledge cutoff are model properties.
Orchestrator agent	The top-level reasoning entity invoked by the harness that interprets the user’s goal, decomposes it into subtasks, selects available tools or subagents, monitors intermediate outputs, and synthesizes the final result.
Reminders	Harness-layer sub-component; the mechanism for re-injecting key instructions at intervals to fight context drift over long-horizon operations. Maintains alignment within an active invocation.
RAG	A grounding approach in which an AI system retrieves external information and inserts it into the model’s context before generating an output or taking a subsequent step. In agentic systems, RAG may occur repeatedly across a workflow, with retrieved material shaping downstream planning, tool use, analysis, and writing. RAG can improve factual grounding, but it does not guarantee accuracy, because errors may arise from incomplete retrieval, poor source ranking, misinterpretation, outdated sources, or compounding downstream reasoning.
Skill	A dynamically invoked capability bundle that extends what the model can effectively do. Includes MCP servers, structured tool definitions, and pre-packaged subagent modules. Skills are invoked by the harness but shape the model's effective capability surface.
Subagent	A reasoning entity invoked by the orchestrator (or by another subagent) to handle a delegated subtask. Each subagent is itself a model + skills + configuration slice, mechanically invoked through tool calls. Subagents may recursively delegate to further subagents.
Tool use	The capacity for a model's outputs to include calls to external software (EHRs, search engines, code interpreters, databases, APIs) whose results feed back into subsequent reasoning steps. This is one of the defining properties of agentic systems. Importantly, tools sometimes rely on deterministic or externally verifiable systems (rather than generative prediction), and so may provide a partial check on language model outputs.

*AI*, Artificial intelligence; *API*, application programming interface; *EHR*, electronic health record; *MCP*, model context protocol; *RAG*, retrieval-augmented generation.

## The ironies of agentic AI

The scientific conversation about AI in research has been, until recently, dominated by the question of whether and how language models should be used to write the text of scientific manuscripts,^2^ how to implement these systems in simple workflows,^3^ and how accurate single-inference runs are at diagnostic or management questions. Yet agentic AI can co-opt the underlying process and workflow that produces the actual scientific hypotheses and data, something far more fundamental to our conception of core scientific inquiry.

Whether such automated loops are truly governable, even by experienced scientists, is an open question, as the inherent impossibility of such oversight—and the potential for broad deskilling—was already highlighted almost a half century ago.^4^^,^^5^ Moreover, the risk of deskilling suggests we may now be at peak oversight: today's clinicians trained before AI was ubiquitous, whereas future generations may be asked to supervise workflows without ever building the underlying skills.

## The infrastructure problem and the category error

As agent adoption becomes more widespread, it is increasingly clear that some challenges are structural vestiges of the first AI revolution rather than hard stops, as agents frequently cannot access the resources their users already have rights to, whether electronic medical record systems or full-text publications. Indeed, an agentic research assistant deployed at a world-class subscribing academic medical center may have less complete access to the medical literature than a high school student with a web browser.

This is not simply an inconvenience; it can lead to a systematic (and underappreciated) degradation in the body of data that research agents can use because these systems rely on abstracts or preprints rather than full-text articles.

Agentic AI access on behalf of an individual user is not the same as allowing data to be used for model training, and conflating the 2 is a category error with profound practical and scientific implications. In model training, articles serve as source material; in access on behalf of a user, articles are accessed and saved for that user’s benefit, and this generally does not directly benefit the AI laboratory. It may be worth rethinking limits on access on behalf of individual users to better mirror the way citation managers are used. Otherwise, we may limit some of the most scientifically and societally valuable uses of AI systems to offset a harm (providing training data) that this access does not actually cause.

## Cancer, speed, and algorithmic feedback loops

The risks and complexity of agentic AI errors increase in proportion to their utility for users. A chatbot that hallucinates produces a single, detectable output. An orchestrator agent that hallucinates can act on its hallucination, call subagents and tools, and produce a slew of downstream artifacts—queries, analyses, code, documents, and orders—that propagate the error across a chain of subsequent steps, making it impossible for even the most responsible user to reconstruct.

The failure modes that matter for agentic AI are thus not related to single-step hallucinations, but to compounding errors across a chain of dependent decisions—a category which the existing literature, with its fetishization of diagnostic accuracy and prediction-model performance, has yet to address rigorously.

An analogy to feedback loops in cancer may be instructive. A chatbot hallucination resembles a transient cellular error that can be cleared locally, while an agentic loop with tool access gone awry more closely resembles a malignancy—a process that, having escaped local regulation, replicates (and compounds) its error, evades oversight, and recruits additional resources to sustain itself. The biological mechanisms that constrain runaway proliferation, such as cell-cycle checkpoints and apoptosis, suggest an alternative conception of agentic safeguards that could be useful beyond medical applications.

It is not that humans do not make mistakes—to err, as we well know in medicine, is human. However, humans cannot make mistakes at the speed and scale of agentic AI systems, and our centuries-old institutional safeguards against errors, from the apprenticeship model to peer review, are calibrated for the relatively modest pace of human error. When the error-generating entity is a fleet of agents acting in parallel, at machine speed, running 24/7, with correlated failure modes propagated by shared weights, our analog governance systems may fail completely.

## Clinical and governance frameworks for the agentic era

The medical conversation about AI needs to move past the chatbot framing. To benefit the scientific enterprise, and ultimately our patients, we need to think carefully and creatively about the higher-order implications of agentic AI and how clinical medicine and scientific research could be restructured.

This conversation must move beyond abstract rules and governance frameworks and acknowledge that agentic AI is already being deployed in the medical context, and its use is already shifting practice and research standards with largely unacknowledged and unstudied feedback loops. Meaningful novel safety approaches—beyond a checkbox nod to a human-in-the-loop, which may be ineffectual once the loop has reshaped the conversation—are needed.

### Declaration of generative AI and AI-assisted technologies in the writing process

During the preparation of this work, the author used OpenAI GPT-5.5 Thinking and Anthropic Claude Opus 4.7 to assist with language editing in the body of the text and table as well as for figure development in vector format. After using these tools, the author reviewed and edited the content as needed and takes full responsibility for the content of the published article.

## Conflicts of interest

None disclosed.

## References

[1] Gottweis J., Weng W.-H., Daryin A. (Published online May 19, 2026). Accelerating scientific discovery with Co-Scientist. Nature.

[2] Kantor J. (2024). The great automatic grammatizator: on the use and misuse of large language models in scientific and academic writing. JAAD Int.

[3] Kantor J. (2024). Best practices for implementing ChatGPT, large language models, and artificial intelligence in qualitative and survey-based research. JAAD Int.

[4] Bainbridge L. (1983). Ironies of automation. Automatica (Oxford).

[5] Arrow K.J. (1962). The economic implications of learning by doing. Rev Econ Stud.

